# Use of Direct Oral Anticoagulants in Patients With Antiphospholipid Syndrome: A Systematic Review and Comparison of the International Guidelines

**DOI:** 10.3389/fcvm.2021.715878

**Published:** 2021-08-03

**Authors:** Daniele Pastori, Danilo Menichelli, Vittoria Cammisotto, Pasquale Pignatelli

**Affiliations:** Department of Clinical, Internal, Anesthesiological and Cardiovascular Sciences, Sapienza University of Rome, Rome, Italy

**Keywords:** vitamin K antagonists, direct oral anticoagulants, antiphospholipid antibody syndrome, guideline, anticoagulants

## Abstract

Antiphospholipid antibody syndrome (APS) requires long-term anticoagulation to prevent recurrent thrombosis. Direct oral anticoagulants (DOACs) have been increasingly used in APS patients, but contradictory guidelines recommendations on their use do exist. We performed a systematic review of literature including studies investigating the role of DOACs in APS patients. At this aim, PubMed and Cochrane databases were searched according to PRISMA guidelines. We identified 14 studies which investigated the use of DOACs in patients with APS, of which 3 randomized clinical trials (RCTs), 1 *post-hoc* analysis of 3 RCTs, 7 case series and 3 cohort studies (2 prospective and 1 retrospective). Among DOACs, rivaroxaban was the most used (*n* = 531), followed by dabigatran (*n* = 90) and apixaban (*n* = 46). Regarding guidelines indications, the 2019 European Society of Cardiology (ESC) and American Society of Hematology (ASH) guidelines recommend against the use of DOACs in all APS patients. The European League Against Rheumatism (EULAR), British Society for Haematology (BSH), and International Society on Thrombosis and Haemostasis (ISTH) guidance provided more detailed indications stating that warfarin should be the first-choice treatment but DOACs may be considered in patients (1) already on a stable anticoagulation with a DOAC, (2) with low-quality anticoagulation by warfarin, (3) unwilling/unable to undergo INR monitoring, (4) with contraindications or serious adverse events under warfarin. Patients with arterial APS or triple positivity should be treated with warfarin while venous APS with single or double positivity may be candidate to DOACs, but high-quality studies are needed.

## Introduction

The incidence and prevalence of antiphospholipid antibody syndrome (APS) are difficult to estimate given that the definition of APS has evolved over the years making epidemiological studies published before 2000 not adhering to the new classification criteria (1). However, a large recent study estimated an incidence of APS of 2.1 per 100,000 per year and a prevalence of 50 per 100,000 inhabitants (2).

APS is an autoimmune disease characterized by the production of auto-antibodies directed against various phospholipids. APS is diagnosed in case of persistent positivity of anticardiolipin (aCL), anti-β2 glycoprotein I (β2GPI), and lupus anticoagulant (LAC) assays, which also play a pathogenic role in determining the risk of thrombotic events (3). However, the persistent positivity to antiphospholipid antibodies (aPL) is not sufficient alone to define APS, which should be accompanied by clinical thrombotic event in the venous and arterial circulation or by obstetrical complications (4). Other non-criteria clinical manifestations in patients with APS include thrombocytopenia, which seems to have a negative prognostic role (5), neurological manifestations (6), and livedo reticularis (7), suggesting that clinical presentation may be heterogeneous and signs/symptoms are not limited to thrombosis.

**Figure 1:**
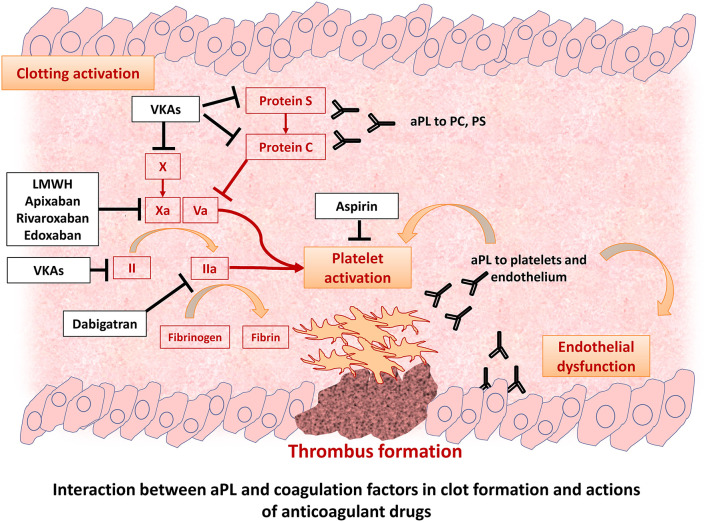
Pathophysiology of thrombotic events in patients with antiphospholipid syndrome.

Thrombotic manifestations are mainly related to the fact that aPL may directly contribute to thrombus formation and platelet activation (Figure 1). Indeed, an increased risk of myocardial infarction (8), ischemic stroke, and peripheral artery disease (9) and neurological disorders in this patient population has been described. After a first thrombotic event, the risk of recurrences sharply increases by 10–67% (10). The thrombotic risk seems to be influenced by the clinical and immunological characteristics of patients with triple positive aPL patients having the highest thrombotic risk, estimated at 5.3% per year (11, 12). However, the thrombotic potential of non-criteria aPL and the value of isolated IgM/LAC is still under investigation (13–15). Furthermore, a significant proportion of patients present a negativization of aPL during follow-up, but it is unclear if it parallels a reduction of thrombotic risk (16).

To reduce the risk of first and recurrent thrombotic events patients with APS require anti-thrombotic treatment. A meta-analysis showed that aspirin administration reduced the risk of first arterial (HR: 0.43, 95%CI 0.20–0.93) but not venous thrombotic event in APS carriers (17). However, after a first thrombotic event, APS patients require long-term treatment with oral anticoagulants. For decades, vitamin K antagonists (VKAs) have represented the only available oral anticoagulant drug. However, some issues regarding the use of VKAs in patients with APS have become evident over time, including the so-called warfarin resistance [i.e., patients needing high weekly amount of VKAs to obtain and maintain therapeutic INR; (18)] and an unstable anticoagulation quality (19). In addition, a significant proportion of patients experience recurrent thrombotic events despite adequate anticoagulation (20), with high-intensity VKA therapy not being superior of standard care in reducing these recurrences (21). Moreover, the addition of aspirin to oral anticoagulation in recurrent arterial APS is still under debate given the lack of clear benefit (22). Finally, adherence to VKA treatment was shown to be progressively reduced over time in different clinical settings (23), with cessation of oral anticoagulation being associated with an increased risk of recurrent thrombotic events in APS (24, 25). For these reasons, adequate anticoagulation therapy still represents a clinical challenge in APS patients.

In the last decade, the direct oral anticoagulants (DOACs) have been increasingly used for the treatment of venous thromboembolism (VTE) and for the thromboprophylaxis of patients with atrial fibrillation. The main advantages of DOACs are the predictable anticoagulant effect, the fixed dose and the rapid onset and offset of action. More recently, the use of DOACs has been tested also in patients with APS with divergent results (26). Aim of this review is to summarize current evidence on the safety and efficacy of DOACs in APS and to compare recommendations provided by international scientific societies.

## Studies Investigating Safety and Efficacy of DOACs in APS Patients

### Information Sources and Search Strategy

We performed a systematic review of literature including studies investigating the role of DOACs in APS patients. At this aim, PubMed and Cochrane databases were searched according to PRISMA guidelines. We included only clinical studies (both observational and randomized clinical trials) involving humans and in English language. Articles with no full text available were also excluded as well as review, commentary, and letters. We used a combination of “antiphospholipid syndrome” and “direct oral anticoagulants” or “apixaban,” “dabigatran,” “edoxaban,” “rivaroxaban.” No time restrictions were applied (last search performed on 27 Jun 2021). The use of “non-vitamin K oral anticoagulants” provided no additional results. Only one study from the same cohort was considered. Case series including <5 patients were excluded.

### Data Collection Process and Data Items

Two physicians (DP and PM) independently screened the titles and abstracts of the manuscripts identified through the database searches to identify studies potentially eligible for further assessment. For each study, we collected the following information: Author (year), study design, follow up (months), triple positivity (%), study sample, type of anticoagulant studied, women (%), age (mean), index event for APS diagnosis, any safety endpoint, any efficacy endpoint.

### Quality Assessment

Quality of included studies was assessed using the National Institutes of Health (NIH) Tools (https://www.nhlbi.nih.gov/health-topics/study-quality-assessment-tools) according to each study type (Table 1): (1) Quality Assessment of Controlled Intervention Studies; (2) Quality Assessment of Controlled Intervention Studies; (3) Quality Assessment Tool for Case Series Studies; (4) Quality Assessment Tool for Observational Cohort and Cross-Sectional Studies.

**Table 1:** Quality assessment for included studies.

**Items**	**RE-COVER(R),** ** RE-COVER II,** ** RE-MEDY (2016)** ^*^ **Goldhaber et al. (27)**	**RAPS (2016)** ^*^ ** Cohen et al. (28)**	**TRAPS (2018)** ^*^ ** Pengo et al. (29)**	**Ordi-Ros et al.** ** (30)** ^*^	**Malec et al.** ** (31)** ^**^	**Malec et al.** ** (32)** ^***^	**Legault et al.** ** (33)** ^***^
1	Y	Y	Y	Y	Y	Y	Y
2	Y	Y	Y	Y	Y	Y	Y
3	NR	N	N	N	Y	NR	Y
4	N	N	N	N	N	Y	Y
5	N	N	N	N	Y	N	Y
6	Y	Y	N	N	Y	Y	Y
7	NR	Y	Y	N	Y	Y	Y
8	NR	Y	Y	Y	N	CD	CD
9	CD	CD	CD	CD	N	Y	Y
10	Y	Y	Y	Y	–	N	N
11	Y	Y	Y	Y	–	Y	Y
12	Y	Y	Y	Y	–	N	N
13	Y	Y	Y	Y	–	NR	Y
14	Y	Y	Y	Y	–	N	N
Total	8/14	10/14	9/14	8/14	6/9	7/14	10/14
**Items**	**Betancur et al**. **(****34**)^**^	**Haladyj and Olesinska** **(** **35** **)** ^**^	**Son et al**. **(****36**)^**^	**Sciascia et al**. **(****37**)^**^	**Noel et al**. **(****38**)^**^	**Resseguier et al**. **(****39**)^**^	**Sato et al**. **(****40**)^**^
1	N	Y	N	Y	Y	Y	Y
2	Y	Y	N	Y	N	Y	Y
3	N	N	Y	N	N	N	NR
4	Y	Y	Y	Y	Y	Y	Y
5	Y	Y	Y	Y	Y	N	N
6	N	N	N	N	Y	Y	N
7	Y	Y	Y	N	Y	Y	Y
8	N	N	N	N	N	Y	CD
9	Y	N	N	N	N	Y	Y
10	–	–	–	–	–	–	N
11	–	–	–	–	–	–	Y
12	–	–	–	–	–	–	N
13	–	–	–	–	–	–	NR
14	–	–	–	–	–	–	Y
Total	5/9	5/9	4/9	4/9	5/9	7/9	7/14

* *Quality Assessment of Controlled Intervention Studies. (1) Was the study described as randomized, a randomized trial, a randomized clinical trial, or an RCT? (2) Was the method of randomization adequate (i.e., use of randomly generated assignment)? (3) Was the treatment allocation concealed (so that assignments could not be predicted)? (4) Were study participants and providers blinded to treatment group assignment? (5) Were the people assessing the outcomes blinded to the participants' group assignments? (6) Were the groups similar at baseline on important characteristics that could affect outcomes (e.g., demographics, risk factors, co-morbid conditions)? (7) Was the overall drop-out rate from the study at endpoint 20% or lower of the number allocated to treatment? (8) Was the differential drop-out rate (between treatment groups) at endpoint 15 percentage points or lower? (9) Was there high adherence to the intervention protocols for each treatment group? (10) Were other interventions avoided or similar in the groups (e.g., similar background treatments)? (11) Were outcomes assessed using valid and reliable measures, implemented consistently across all study participants? (12) Did the authors report that the sample size was sufficiently large to be able to detect a difference in the main outcome between groups with at least 80% power? (13) Were outcomes reported or subgroups analyzed prespecified (i.e., identified before analyses were conducted)? (14) Were all randomized participants analyzed in the group to which they were originally assigned, i.e., did they use an intention-to-treat analysis?*

** *Quality Assessment Tool for Case Series Studies. (1) Was the study question or objective clearly stated? (2) Was the study population clearly and fully described, including a case definition? (3) Were the cases consecutive? (4) Were the subjects comparable? (5) Was the intervention clearly described? (6) Were the outcome measures clearly defined, valid, reliable, and implemented consistently across all study participants? (7) Was the length of follow-up adequate? (8) Were the statistical methods well-described? (9) Were the results well-described?*

*** *Quality Assessment Tool for Observational Cohort and Cross-Sectional Studies. (1) Was the research question or objective in this paper clearly stated? (2) Was the study population clearly specified and defined? (3) Was the participation rate of eligible persons at least 50%? (4) Were all the subjects selected or recruited from the same or similar populations (including the same time period)? Were inclusion and exclusion criteria for being in the study prespecified and applied uniformly to all participants? (5) Was a sample size justification, power description, or variance and effect estimates provided? (6) For the analyses in this paper, were the exposure(s) of interest measured prior to the outcome(s) being measured? (7) Was the timeframe sufficient so that one could reasonably expect to see an association between exposure and outcome if it existed? (8) For exposures that can vary in amount or level, did the study examine different levels of the exposure as related to the outcome (e.g., categories of exposure, or exposure measured as continuous variable)? (9) Were the exposure measures (independent variables) clearly defined, valid, reliable, and implemented consistently across all study participants? (10) Was the exposure(s) assessed more than once over time? (11) Were the outcome measures (dependent variables) clearly defined, valid, reliable, and implemented consistently across all study participants? (12) Were the outcome assessors blinded to the exposure status of participants? (13) Was loss to follow-up after baseline 20% or less? (14) Were key potential confounding variables measured and adjusted statistically for their impact on the relationship between exposure(s) and outcome(s)?*

*CD, cannot be determined; NA, not applicable; NR, not reported; N, no; Y, yes*.

### Study Characteristics and Quality Evaluation

**Figure 2:**
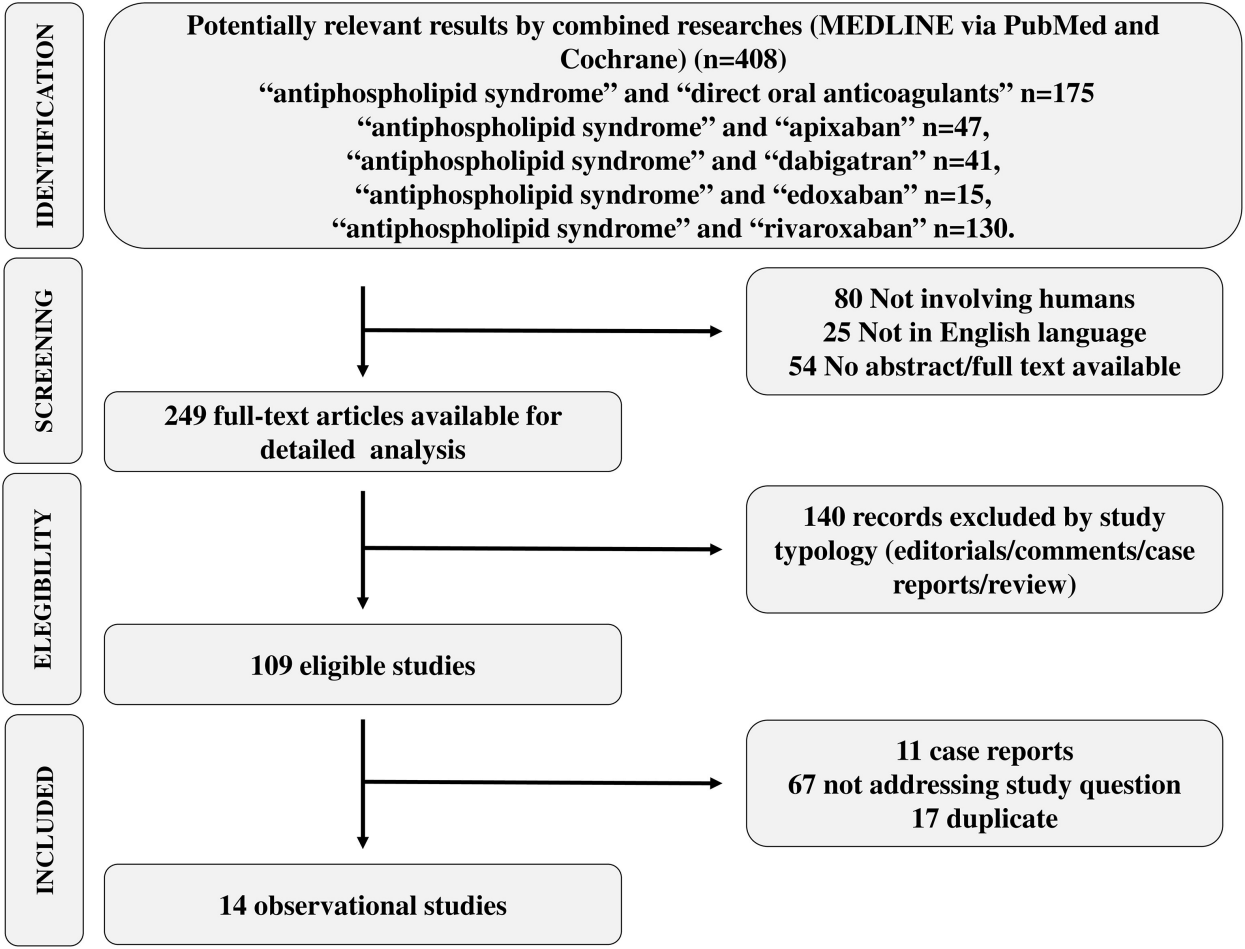
PRISMA flow chart.

Strategy search and reasons for exclusion are reported in Figure 2. Table 2 reports clinical studies on the safety and efficacy of DOACs in APS patients. We identified 14 studies which investigated the use of DOACs in patients with APS, of which 3 randomized clinical trials (RCTs), 1 *post-hoc* analysis of 3 RCTs, 7 case series and 3 cohort studies (2 prospective and 1 retrospective) (Table 2). Quality evaluation showed that the quality of RCT and *post-hoc* of RCT ranged from 8/14 to 10/14 mainly due to lack of blindness in treatment allocation, that is however, intrinsic in this type of studies comparing a dose-adjusted to a fixed-dose treatment (Table 1). The quality of case series was generally 4–5/9 with only two studies scoring 6/9 (31) and 7/9 (39) (Table 1). These results are essentially due to a poor description of statistical methods (some of these series were published in form of brief report or letter) and lack of consecutive recruitment of patients (Table 1).

**Table 2:** Characteristics of studies enrolling patients with APS treated with DOACs.

**Author** ** (year)**	**Design**	**Follow up** ** (months)**	**Triple** ** positive (%)**	**Study** ** sample**	**Anticoagulant**	**Women** ** (%)**	**Age** ** (mean)**	**Index** ** event**	**Safety** ** endpoint**	**Efficacy** ** endpoint**
RE-COVER(R), RE-COVER II, RE-MEDY (2016) (27)	*Post-hoc* RCTs	NR	NR	151	Dabigatran: 71 VKA: 80	36.4	47.6	VTE	MB (ISTH criteria), CRB and any bleeding **Results:** Similar MB and CRBs. Less any bleeding with dabigatran (HR 0.50, 95%CI 0.26–0.95)	Recurrent VTE/VTE-related death **Results:** Similar VTE between dabigatran and warfarin (HR 0.43, 95%CI 0.08–2.38)
RAPS (2016) (28)	RCT	7.0	28.0	116	Rivaroxaban: 57 VKA: 59	72.4	48.5	VTE	MB, CRB, and minor bleedings **Results:** No MB or CRB occurred	Thromboembolism **Results:** No thrombotic events occurred
TRAPS (2018) (29)	RCT	20.4	100.0	120	Rivaroxaban: 59 VKA: 61	64.2	46.3	Arterial, venous, and/or biopsy-proven micro-thrombosis.	Arterial or venous thromboembolic events, MB, and vascular death **Results:** 13 total events (7 thrombotic and 6 MB): 11 (19%) in the rivaroxaban and 2 (3%) in the warfarin group Rivaroxaban: 4 IS and 3 MI, and 4 (7%) MB Warfarin: no thrombotic events and 2 (3%) MB. No death reported	
Ordi-Ros et al. (30)	RCT	36.0	60.5	190	Rivaroxaban: 95 VKA: 95	63.7	49.0	Arterial or venous thrombosis	MB **Results:** MB occurred in 6 patients (6.3%) in the rivaroxaban group and 7 (7.4%) in the VKA group (RR 0.86, 95%CI 0.30–2.46)	Venous and arterial thrombosis **Results:** 11 recurrent thrombosis in the rivaroxaban and 6 in the VKA group (RR 1.83, 95%CI, 0.71–4.76) More IS with rivaroxaban (RR 19.00, 95%CI, 1.12–321.9)
Malec et al. (31)	P Case series	22.0	28.6	56	Rivaroxaban: 49 Dabigatran: 4 Apixaban: 3	78.6	52.0	VTE	MB according to ISTH criteria **Results:** 2 severe bleedings	VTE **Results:** 6 (10.7%) VTE (5.8%/year)
Malec et al. (32)	P	51.0	26.1	176	Rivaroxaban: 36 Dabigatran: 4 Apixaban: 42 VKA: 94	83.0	44.5	VTE or arterial thrombosis	MB or CRB **Results:** DOACs increased risk of MB or CRNMB if menstrual bleeding were included (HR 3.63, 95%CI 1.53–8.63) GI bleeds and MB or CRNMB other than menstrual bleeding were similar between groups	Composite of VTE, cerebrovascular ischemic events or MI **Results:** Increased thrombosis with DOACs (HR 3.98, 95%CI 1.54–10.28) and recurrent VTE (HR 3.69, 95%CI 1.27–10.68) compared with VKAs
Legault et al. (33)	P	19.0	0.0	82	Rivaroxaban	47.6	53.4	VTE	MB Minor bleeding **Results:** There were no MB but 23 minor bleeding occurred	VTE, myocardial infarction, IS, and cardiovascular death **Results:** 4 thrombotic events (2 cerebrovascular and 2 VTE)
Betancur et al. (34)	Case series	19.0	12.5	8	Rivaroxaban: 7 Apixaban: 1	100.0	45.5	VTE (87.5%), PE (62.5%), and arterial thrombosis (75%), 25% obstetrical	–	Recurrence of thrombosis **Results:** There was no recurrence of thrombosis
Haladyj and Olesinska (35)	P Case series	20.0	17.4	23	Rivaroxaban	100.0	NR	8 arterial thrombosis, 9 VTE, 5 both	MB and minor bleeding **Results:** No MB or minor bleeding occurred	Arterial or venous thrombosis **Results:** 1 arterial thrombosis
Son et al. (36)	P Case series	11.4	41.7	12	Rivaroxaban	58.3	42.0	VTE and/or IS	–	Recurrent DVT **Results:** 2 patients had recurrent DVT
Sciascia et al. (37)	P Case series	10.0	NR	35	Rivaroxaban	68.6	47.0	Previous DVT (n: 24) and 11 DVT and PE	MB **Results:** No MB occurred	VTE **Results:** No VTE occurred
Noel et al. (38)	R Case series	19.0	26.9	26	Rivaroxaban: 15 Dabigatran: 11	53.8	39.1	Arterial and/or venous thrombosis, pregnancy morbidity	Bleeding events **Results:** 2 bleedings under Rivaroxaban: one hyper-menorrhea and one rectal bleeding	Thrombotic recurrence **Results:** One cutaneous microthrombosis under Rivaroxaban
Resseguier et al. (39)	R Case series	35.6	8.7	23	Rivaroxaban	56.5	41.0	VTE (*n*: 19), artery event (*n*: 2) or both (*n*: 1), and catastrophic APS (*n*: 1)	MB **Results:** No MB occurred	Arterial and venous thrombotic events **Results:** One patient developed PE
Sato et al. (40)	R	5 years	33.3	206	Factor Xa Inhibitors: 18 Warfarin: 36	86.0	42.8	34 arterial 32 VTE 11 pregnancy morbidity	Severe bleeding requiring hospitalization and/or blood transfusion **Results:** 1 and 2 cases of recurrences of thrombosis in the factor Xa Inhibitors and warfarin groups, respectively	Arterial/venous thrombosis **Results:** 6 and 8 cases of recurrences of thrombosis in the factor Xa Inhibitors and warfarin groups, respectively

*CI, confidence interval; CRB, clinical relevant bleeding; CRNMB, clinical relevant non-major bleeding; DOAC, direct oral anticoagulant; DVT, deep vein thrombosis; GI, gastrointestinal; HR, hazard ratio; IS, ischemic stroke; ISTH, International Society on Thrombosis and Haemostasis; MI, myocardial infarction; MB, major bleeding; NR, not reported; P, prospective; PE, pulmonary embolism; R, retrospective; RCT, randomized clinical trial; RR, relative risk; VKA, vitamin K antagonist; VTE, venous thromboembolism*.

Regarding the 3 cohort studies, they generally lacked a formal sample size justification, blind adjudication of event, exposure assessment only at baseline and did not report the rate of patients lost during follow-up (Table 1).

Women represented the majority of patients among the studies, and the mean age of the population range between 39.1 and 53.4 years. Clinical events for the initiation of anticoagulation were mainly represented by venous thromboembolism, but two RCTs included both arterial and venous thrombosis as clinical index event. Two studies included also patients with obstetrical APS (34, 35), however, DOACs are not recommended in obstetrical APS and in lactating women, as they have a variable excretion rate in human milk and data on their safety are still lacking (41).

Among DOACs, rivaroxaban was the most represented with 531 treated patients, followed by dabigatran with 90 patients and apixaban with 46 patients. All RCTs (28–30) compared rivaroxaban with VKAs, while a *post-hoc* analysis of RE-MEDY and RE-COVER trials compared dabigatran with VKAs (27).

### Clinical Outcomes

The follow-up ranged from 7 to 5 years (Table 2). The efficacy endpoints were the recurrence of VTE or a composite of arterial and venous thrombosis; safety endpoints were major or clinically relevant bleedings.

In two RCTs (29, 30) rivaroxaban was associated with an increased risk of thrombotic events without an increased risk of bleeding. Of note, these studies included APS patients with both arterial and venous thrombotic events and a high proportion of patients with triple positivity.

The only study which showed an increased risk of bleeding included mostly APS women (>80%) with a high rate of heavy menstrual bleeding (HMB); while, no differences between two groups were reported regarding major, gastrointestinal or clinical relevant non-major bleeding (CRNMB) (32).

A *post-hoc* analysis of RE-MEDY and RE-COVER which compared dabigatran to VKAs (27) in patients with inherited disorders of whom APS represented the second most common thrombophilia accounting for 20% of all patients (27), and a RCT (28), which compared rivaroxaban and VKAs showed similar safety and efficacy profiles between DOACs and VKAs (Table 2).

## Guidelines Recommendations/Consensus Suggestions

Based on the above discussed studies, different international thrombosis and cardiology societies provided discordant recommendations on the use of DOACs in patients with APS.

The grading system used to provide the level of evidence differed among guidelines and are reported in the footnote of the Table 3.

**Table 3:** International guideline recommendations/consensus suggestions on the use of DOACs in APS patients.

**Guidelines**	**Recommendations**	**Level of evidence**
**International guidelines on deep vein thrombosis/pulmonary embolism**		
ESC 2019 (42)	Indefinite treatment with a VKA is recommended for patients with APS	Ib^*^
	DOACs are not recommended in patients with severe renal impairment, during pregnancy and lactation, and in patients with APS	IIIc^*^
ASH 2020 (43)	For patients with DVT and/or PE, the ASH guideline panel suggests using DOACs over VKAs (conditional recommendation based on moderate certainty in the evidence of effects). *Remarks: This recommendation may not apply to certain subgroups of patients, such as those with renal insufficiency (creatinine clearance, 30 mL/min), moderate to severe liver disease, or APS*	Remark. Evidence not provided
NICE 2020 (44)	Offer people with confirmed proximal deep vein thrombosis or pulmonary embolism and an established diagnosis of triple positive APS LMWH concurrently with a VKA for at least 5 days, or until the INR is at least 2.0 in two consecutive readings, followed by a VKA on its own	^∧^
**International guidelines on antiphospholipid syndrome**		
BSH Guidelines 2020 (45)	*Patients with arterial thrombosis* For anticoagulation for treatment and secondary prophylaxis of arterial thrombosis in patients with APS, we recommend VKAs and do not recommend DOACs	IB^#^
	*Patients with triple positive APS and venous thrombosis* We recommend against the initiation of DOACs for treatment or secondary prophylaxis in patients with venous thrombosis and known triple positive APS. For patients with triple positive APS who are currently on a DOAC, we recommend switching from the DOAC to a VKA after discussion with patients regarding the available evidence. *For those patients who do not wish to switch, we recommend continuation of the DOAC over no anticoagulation*	IB^#^
	*Patients with non-triple positive APS and venous thrombosis* There is insufficient evidence to make strong recommendations in this group of patients. We suggest against the initiation of DOACs for treatment or secondary prophylaxis in patients with venous thrombosis and known non-triple positive APS. *Patients who are already on a DOAC may continue or switch to a VKA after discussion with the patient taking into account their clinical history, treatment adherence and previous experience. For those patients who do not wish to switch, we recommend continuation of the DOAC over no anticoagulation*	IIC^#^
ISTH 2020 guidance (46)	*We recommend that for the treatment of thrombotic APS among patients with any of the following (termed “high-risk” APS patients)* (a) triple positivity, (b) arterial thrombosis, (c) small vessel thrombosis or organ involvement (d) heart valve disease according to Sydney criteria, VKA should be used instead of DOACs	Not provided
	We recommend that DOACs should not be used in APS patients with recurrent thrombosis while on therapeutic intensity VKA. In this circumstance, other therapeutic options may include an increased target INR range, treatment dose LMWH, or the addition of antiplatelet therapy	Not provided
	We recommend that DOACs should not be used in APS patients who are non-adherent to VKA. In this circumstance, other options may include education on adherence to VKA treatment along with frequent INR testing	Not provided
	In single or double positive non- “high risk” APS patients who have been on DOACs with good adherence for several months for a first episode of VTE, we recommend a discussion with the patient of options including perceived risks and uncertainties, in the spirit of shared decision-making and review of whether continued treatment with a DOAC is appropriate	Not provided
	In single- or double-positive non- “high-risk” APS patients with a single prior VTE requiring standard-intensity VKA, with allergy or intolerance to VKA or erratic INRs despite patient adherence, we suggest that alternative VKAs, if available, should be considered prior to consideration of a DOAC	Not provided
EULAR 2019 (47)	*In patients with definite APS and first venous thrombosis:* Rivaroxaban should not be used in patients with triple aPL positivity due to the high risk of recurrent events	1b/B^§^
	*In patients with definite APS and first venous thrombosis:* DOACs could be considered in patients not able to achieve a target INR despite good adherence to VKA or those with contraindications to VKA (e.g., allergy or intolerance to VKA)	5/D^§^
	*In patients with definite APS and first arterial thrombosis:* Rivaroxaban should not be used in patients with triple aPL positivity and arterial events	Ib/B^§^
	*In patients with definite APS and first arterial thrombosis:* Based on the current evidence, we do not recommend use of DOACs in patients with definite APS and arterial events due to the high risk of recurrent thrombosis	5/D^§^

*APS, antiphospholipid antibody syndrome; ASH, American Society of Hematology; BSH, British Society for Haematology; DOAC, direct oral anticoagulant; DVT, deep vein thrombosis; ESC, European Society of Cardiology; EULAR, European League Against Rheumatism; INR, international normalized ratio; ISTH, International Society on Thrombosis and Haemostasis; LMWH, low-molecular weight heparin; NICE, National Institute for Health and Care Excellence; PE, pulmonary embolism; VKA, vitamin K antagonist; VTE, venous thromboembolism*.

* *ESC Committee for Practice Guidelines (CPG) policy*.

§ *Oxford Centre for Evidence-Based Medicine standards*.

# *Grading of Recommendations Assessment, Development and Evaluation (GRADE)*.

∧ *Based on the Medicines and Healthcare products Regulatory Agency alert and the experience and opinion of the Guideline Committee*.

The 2019 European Society of Cardiology (ESC) guidelines recommend against the use of DOACs in APS patients, with no distinction among different DOACs, or between venous and arterial APS or among single, double or triple positive patients (Table 3). This recommendation seems however, to be based only on the results of the Trial on Rivaroxaban in AntiPhospholipid Syndrome (TRAPS) trial (29), which included triple positive thrombotic APS patients with both previous venous and arterial events, randomized to receive Rivaroxaban or conventional treatment. However, it should be noted that treatment of arterial events is not an indication to DOAC treatment. No mention is therefore given in case of venous or single/double positive APS patients. The results of this trial using Rivaroxaban were applied to all other DOACs.

Similarly, in the 2020 International Society on Thrombosis and Haemostasis (ISTH) guidance (46), DOACs are not considered as a valid option for any APS patient, with the only possibility of continuing DOAC in stable low-risk patients already on treatment, after shared informed discussion.

A more detailed indication on the use of DOACs is provided by the 2019 European League Against Rheumatism (EULAR) guidelines (47), which have taken into consideration the clinical phenotype of APS patients based on the presence of a venous or arterial event as indication to anticoagulation (Table 3). Thus, while DOACs and in particular Rivaroxaban, are contraindicated in APS patients with triple aPL positivity and/or an arterial event, its use may be considered in venous APS patients without triple aPL positivity (47). Another important difference between ESC and EULAR guidelines is that the latter consider the possibility that patients on VKAs may have low-quality therapy (i.e., low time in therapeutic range, TiTR) or may be intolerant to VKA treatment. In these cases, the use of DOACs may be considered (47).

A similar approach has been proposed by the 2020 British Society of Haematology (BSH) Guidelines, which suggest against the use of DOACs in arterial APS patients. In venous APS patients both triple and non-triple who are already on treatment with DOACs, treatment may be continued if patients refuse to switch to VKAs (45).

The 2020 American Society of Hematology (ASH) guidelines (43) state that APS patients are not optimal candidate for DOAC treatment, and suggest the use of low molecular weight heparin (LMWH) over DOAC in case of recurrent event under VKAs. However, the Authors acknowledge that this recommendation is based on very low certainty of the evidence of effects.

## Discussion/Observations

This systematic review of clinical studies showed that the safety and efficacy of DOACs may be highly dependent on clinical and immunological phenotype of APS patients. Of note, none of the studies including non-triple venous APS patients reported an excess of thrombotic recurrence, which was conversely more evident in studies including triple positive or arterial APS patients. It is therefore important to identify the clinical phenotype of patients with APS to establish in which subgroup the use of DOACs may be beneficial. In this context, a recent meta-analysis confirmed this approach showing a four-fold higher thrombotic risk in APS patients with triple positivity (56 vs. 23%; OR = 4.3, 95%CI 2.3–7.7, *p* < 0.0001) as well as in patients with a history of arterial thrombosis (32 vs. 14%; OR = 2.8, 95%CI 1.4–5.7, *p* = 0.006) on treatment with DOACs (12).

**Figure 3:**
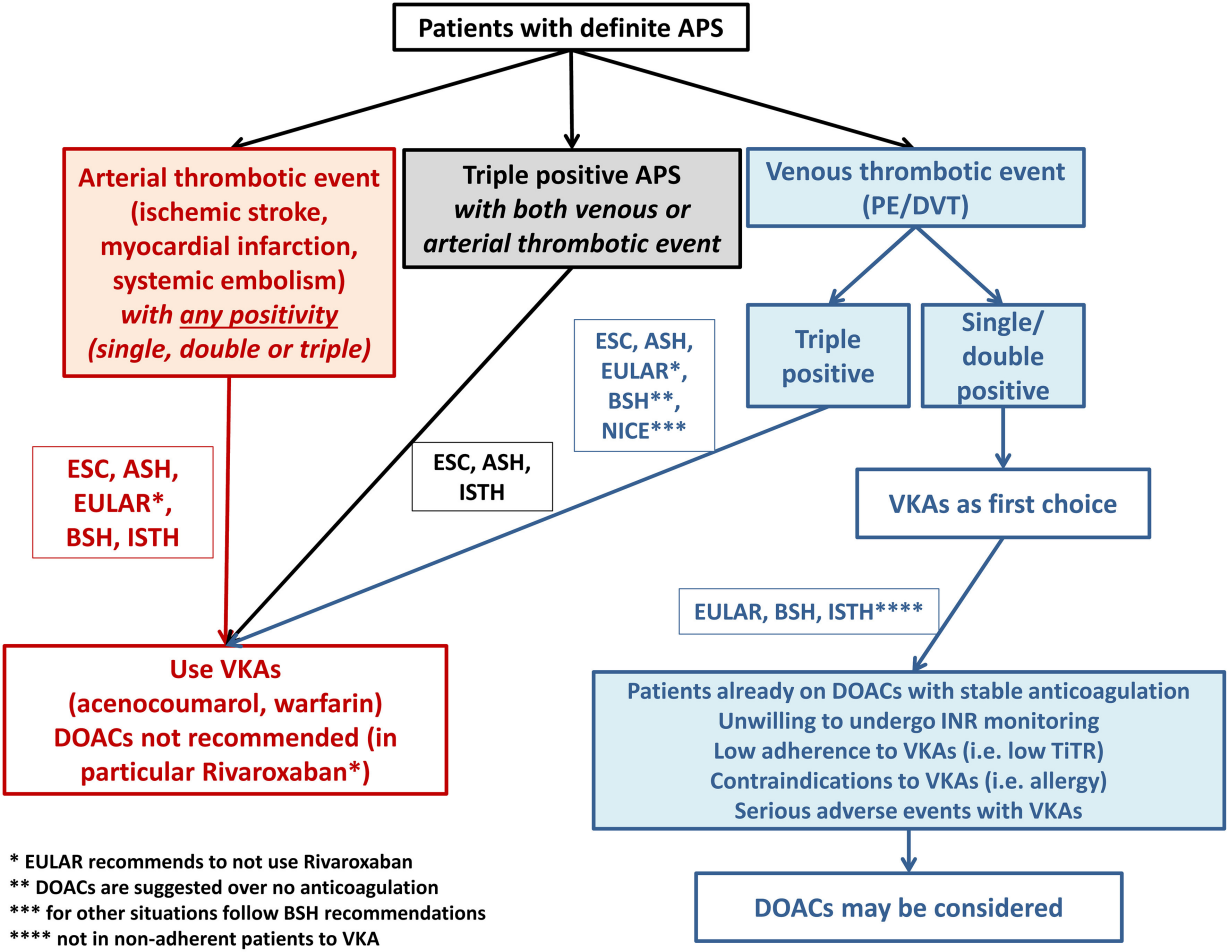
Summary of guidelines recommendations on anticoagulant treatment prescription in patients with antiphospholipid syndrome.

The results from these studies have been differently received by expert committees of international societies to provide clinical recommendations on the use of DOACs in this patient population. Figure 3 summarizes current indications provided by international guidelines on the use of oral anticoagulants in patients with APS. While there is a general agreement on the contraindication on the use of DOACs, and in particular rivaroxaban, in patients with arterial APS and/or triple positivity, there are some differences regarding venous and non-triple APS patients.

Thus, while the ESC and ASH guidelines do not recommend the use of DOACs in any APS patients (with no level of evidence reported in the latter), there was an effort from EULAR, BSH, and ISTH to take into consideration the clinical phenotype of patients for choosing the most appropriate anticoagulant drug (Figure 3). These societies state that VKAs should always represent the first-choice treatment in venous non-triple APS but open to the possibility of using DOACs in some specific situations and in any cases after a shared informed decision with the patient. In particular, patients diagnosed with APS after VTE but who are already on a stable anticoagulation with a DOAC may be kept on the same treatment, as the benefit of switching to VKAs may not be evident in this case. Similarly, patients with very low-quality anticoagulation by VKAs (i.e., TiTR <60%), experiencing INR instability and needing frequent INR checks may benefit more from a stable anticoagulation provided by fixed dose DOAC. Another group potentially suitable for DOAC treatment is represented by patients unwilling or unable to undergo INR monitoring as in the case of difficult access to healthcare facilities or impaired mobility, as treatment with DOAC may be beneficial over not treatment. Finally, patients with contraindications (i.e., allergy) or serious adverse events under VKA therapy may be considered for DOAC treatment. However, it should be noted that the indications provided by the ISTH is based on an expert consensus and no level of evidence for such recommendations is given.

Regarding the type of DOAC, rivaroxaban has been the most widely investigated drug, while the number of patients treated with dabigatran or apixaban is still low. A randomized trial investigating the efficacy and safety of Apixaban in APS patients is currently ongoing and has been modified to exclude patients with arterial thrombosis based on literature data (48); however, this study is actually closed. Patients who were enrolled are still being followed, although it is unclear if they are still being maintained on apixaban or not. No data regarding the use of edoxaban in this patient population are available.

Although DOACs do not require laboratory monitoring to ascertain their efficacy, the assessment of blood concentration of DOACs may turn particularly useful for patients with APS to verify if appropriate peak and trough concentrations are obtained after the drug administration. These values have been shown to correlate with bleeding or thrombotic complications (49). In this context, previous evidence showed that the twice-daily dosing regimens with Apixaban and Dabigatran are associated with less high peak or low trough concentrations (50). More importantly, these twice-daily drugs might guarantee a more stable anticoagulation level in APS patients, leaving patients less exposed to low trough concentrations which are associated with thrombotic events (51).

In conclusion, international guidelines agree on the exclusive use of VKAs in patients with arterial APS and triple positivity (Figure 3). Evidence on venous APS is weak and patients with single or double positivity may be candidate to DOACs, after a shared informed decision with patients, especially in patients who are not willing or have contraindications to VKAs. The lack of consensus among guidelines/consensus originate from the paucity of randomized studies and the lack of rigorous patients' stratification.

## Author Contributions

DP and DM: conceptualization and draft of the manuscript. VC and PP: design and revision of the manuscript. All authors provide approval for publication of the content.

## Conflict of Interest

The authors declare that the research was conducted in the absence of any commercial or financial relationships that could be construed as a potential conflict of interest.

## Publisher's Note

All claims expressed in this article are solely those of the authors and do not necessarily represent those of their affiliated organizations, or those of the publisher, the editors and the reviewers. Any product that may be evaluated in this article, or claim that may be made by its manufacturer, is not guaranteed or endorsed by the publisher.
